# Atypical chemokine receptor ACKR2-V41A has decreased CCL2 binding, scavenging, and activation, supporting sustained inflammation and increased Alzheimer’s disease risk

**DOI:** 10.1038/s41598-020-64755-1

**Published:** 2020-05-15

**Authors:** Josue D. Gonzalez Murcia, Allen Weinert, Claudia M. Tellez Freitas, Daniel K. Arens, Meganne N. Ferrel, Julianne H. Grose, Perry G. Ridge, Eric Wilson, John S. K. Kauwe, K. Scott Weber

**Affiliations:** 1 Brigham Young University, Department of Biology, 4102 Life Sciences Building, Provo, Utah 84602 USA; 2 Brigham Young University, Department of Microbiology and Molecular Biology, 4007 Life Sciences Building, Provo, Utah 84602 USA; 3 Roseman University of Health Science College of Dental Medicine 10894 S, River Front Parkway, South Jordan, UT 84095 USA

**Keywords:** Cellular neuroscience, Genetics of the nervous system, Molecular neuroscience, Neuroimmunology

## Abstract

A recent genome-wide association study (GWAS) of 59 cerebrospinal fluid (CSF) proteins with a connection to Alzheimer’s disease (AD) demonstrated an association between increased levels of chemokine ligand 2 (CCL2) with an atypical chemokine receptor chemokine-binding protein 2 variant V41A (ACKR2-V41A; rs2228467). High levels of CCL2 are associated with increased risk of AD development as well as other inflammatory diseases. In this study we characterized the biological function of the ACKR2-V41A receptor compared to the wild type allele by measuring its ligand binding affinity, CCL2 scavenging efficiency, and cell activation sensitivity. We transfected Chinese hamster ovary cells with plasmids carrying wild type ACKR2 (ACKR2-WT) or the mutant ACKR2-V41A receptor. Binding affinity assays showed that ACKR2-V41A has a lower binding affinity for CCL2 and CCL4 than ACKR2-WT. CCL2 scavenging results aligned with binding affinity assays, with ACKR2-V41A cells scavenging CCL2 with a lower efficiency than ACKR2-WT. Cell activation assays also showed that ACKR2-V41A cells had significantly lower receptor upregulation (β-Arrestin-dependent signaling pathway) upon stimulation compared to ACKR2-WT cells. These findings provide molecular and biological mechanistic insights into the GWAS association of ACKR2-V41A with increased levels of CCL2 in CSF and possibly other chemokine ligands. Increased CCL2 levels are associated with accelerated cognitive decline and increased risk of AD. Understanding how this atypical chemokine receptor allele increases serum markers of inflammation could lead to novel therapeutic solutions for AD.

## Introduction

Inflammation is associated with increased risk of developing cancer, autoimmune disorders, and neurodegenerative diseases such as Alzheimer’s disease (AD)^1^. Acute inflammation plays an essential role in the normal response to tissue injury^2,3^. This inflammatory response initiates a cascade of cellular activation signals in innate immune cells (e.g. macrophages, mast cells, and endothelial cells), resulting in increased production of proinflammatory cytokines and chemokines^4–6^. These cytokines and chemokines are essential to the recruitment and activation of other cells in the innate and adaptive immune systems. An inappropriate response to inflammation or alterations in the production of these chemokines can result in disease development.

AD is the most common neurodegenerative disorder and is the main cause of dementia in the elderly population^7^. Significant progress has been made towards understanding the genetic architecture of AD. To date, 32 loci have been identified with common and rare alleles that influence the risk of AD^8–10^. AD is pathologically characterized by the accumulation of amyloid beta (Aβ) peptide forming insoluble aggregated plaques in the gray matter and is thought to result in neurodegeneration and loss of memory function^5,6,11^. However, the characterization of biological mechanisms by which these rare variants and the accumulation of Aβ modulate risk for disease has proven to be extremely difficult, with only several of these variants being experimentally characterized^8,12–19^.

Recent analyses of quantitative AD endophenotypes in cerebrospinal fluid (CSF) has identified a chemokine receptor variant that alters the risk and rate of progression of AD^20^. This genome-wide association study (GWAS) of 59 AD-related CSF analytes used two independent datasets: the Knight Alzheimer’s Disease Research Center (ADRC) and Alzheimer’s Disease Neuroimaging Initiative (ADNI)^20^. This study demonstrated an association between increased levels of chemokine ligand 2 (CCL2) with atypical chemokine receptor chemokine-binding protein 2 (ACKR2) variant V41A.

CCL2 is also known as monocyte chemotactic protein-1 (MCP-1) and is encoded by the *CCL2* gene located on chromosome 17q11.2-q12. CCL2 is a pro-inflammatory chemokine involved in recruitment of immune cells from the blood to sites of inflammation via chemokine gradients. CCL2 helps to control blood brain barrier migration of monocytes and dendritic cells and aids in the differentiation and migration of macrophages^21^. In the brain, CCL2 is mainly secreted by astrocytes, microglia, and in low levels by endothelial cells^22^. Upon an immune response to infection, injury or inflammation, CCL2 is produced in the central nervous system (CNS)^23^. CCL2 interacts with chemokine receptor CCR2 which signals via a G couple protein cascade. Upon CCR2 and CCL2 ligand interaction, a cascade of cell activation events takes place [i.e. activation of protein kinase C (PKC), calmodulin-dependent protein kinase II (CaMKII), PI3K, Akt, and ERK]. This activation cascade signals cell migration, cell survival, transcription regulation, and release of pro-nociceptive molecules^24,25^. Upon CCL2 binding to a receptor, it induces a strong chemotactic response and mobilization of intracellular calcium ions and synaptic network activity in the hippocampal neurons^26,27^. High levels of CCL2 is a risk factor in several neuroinflammatory and neurodegenerative brain diseases such as multiple sclerosis, brain ischemia, traumatic brain damage, and AD^28^. In AD mouse models, CCL2 is key to inducing chronic inflammation and activation of immune cells, and secretion of other chemokines^29^. Overexpression of CCL2 retains activated microglia cells near the inflamed site^30^. This results in increased interactions of activated microglia with key features of AD: amyloid plaques, plague aggregation, and cognitive decline^31–33^. Inhibition or removal of CCL2 in AD mouse models revealed accelerated amyloid pathology formation^34^. These studies demonstrate that proper function and regulation of CCL2 is imperative to preserve brain innate immune response homeostasis and cognitive function.

Atypical chemokine receptor 2 (ACKR2^35^) is encoded by the *ACKR2* gene located on chromosome 3p21.3. ACKR2 is a seven transmembrane G-protein-coupled receptor containing three intracellular loops and three extracellular loops^36^. ACKR2 binds with the 14 inflammatory chemokines of the CC family, including CCL2 and CCL4, but not CCL19^37^. ACKR2 is an atypical chemokine due to its lack of a canonical DRYLAIV motif in the second extracellular loop. This lack of motif inhibits the ability of the receptor to signal the production of other cytokines^36^. Instead, ACKR2 recognizes inflammatory chemokines, scavenges them, and upregulates the production of more ACKR2 receptor^38^. ACKR2 plays an essential role in the regulation of the inflammatory response by internalizing inflammatory chemokines, facilitating their destruction when the cellular endosome fuses with an acidic lysosome. The chemokine-free ACKR2 receptor is then recycled back to the cell surface and can scavenge additional inflammatory chemokines^39^. ACKR2 is mainly expressed on leukocytes, including dendritic cells, monocytes, macrophages, and innate-like B cells^40^. In the central nervous system (CNS) ACKR2 is mainly expressed in microglia and astrocyte cells^41–43^. Nonsynonymous mutations mapped to the *ACKR2* gene have been associated with increased risk for breast cancer, testicular leukemia, CD45 deficiency, glucose intolerance and inhibit leukocyte type differentiations and monocyte counts^44–49^.

The ACKR2-V41A allele (rs2228467) is associated with altered chemokine levels in the CSF and bloodstream. A 2017 study with 8,293 Finn participants identified increased levels of the chemokine eotaxin (a potent eosinophil attractant) associated with rs2228467 and increased risk of developing Crohn’s disease, multiple sclerosis, and ulcerative colitis^50^. In a different study involving over 11,000 subjects from the electronic Medical Records and Genomics Network (eMERGE), rs2228467 was associated with increased coronary heart disease and a higher monocyte count in the blood stream^44,51^. Rs2228467 was also identified as an increased risk factor for developmental disorders following the analysis of over 700 mother-infant pair mid-gestational serum and neonatal bloodspots^52^. Another 2017 study using the National Human Genome Research Institution (NHGRI) dataset found that rs2228467 is associated with increased AD risk^53^. Together these studies make a compelling case for the biological characterization of ACKR2-V41A. Here we provide the molecular and biological characterization of ACKR2-V41A and show that it has decreased binding affinity, scavenging efficiency, and receptor upregulation (β-Arrestin-dependent signaling pathway) for CCL2. These results provide insights into the previously reported association of ACKR2-V41A with AD risk, rate of cognitive decline, and serum markers of inflammation which could lead to novel therapeutic solutions for AD.

## Results

### ACKR2-V41A has similar predicted protein structure compared to wild type

**Figure 1:**
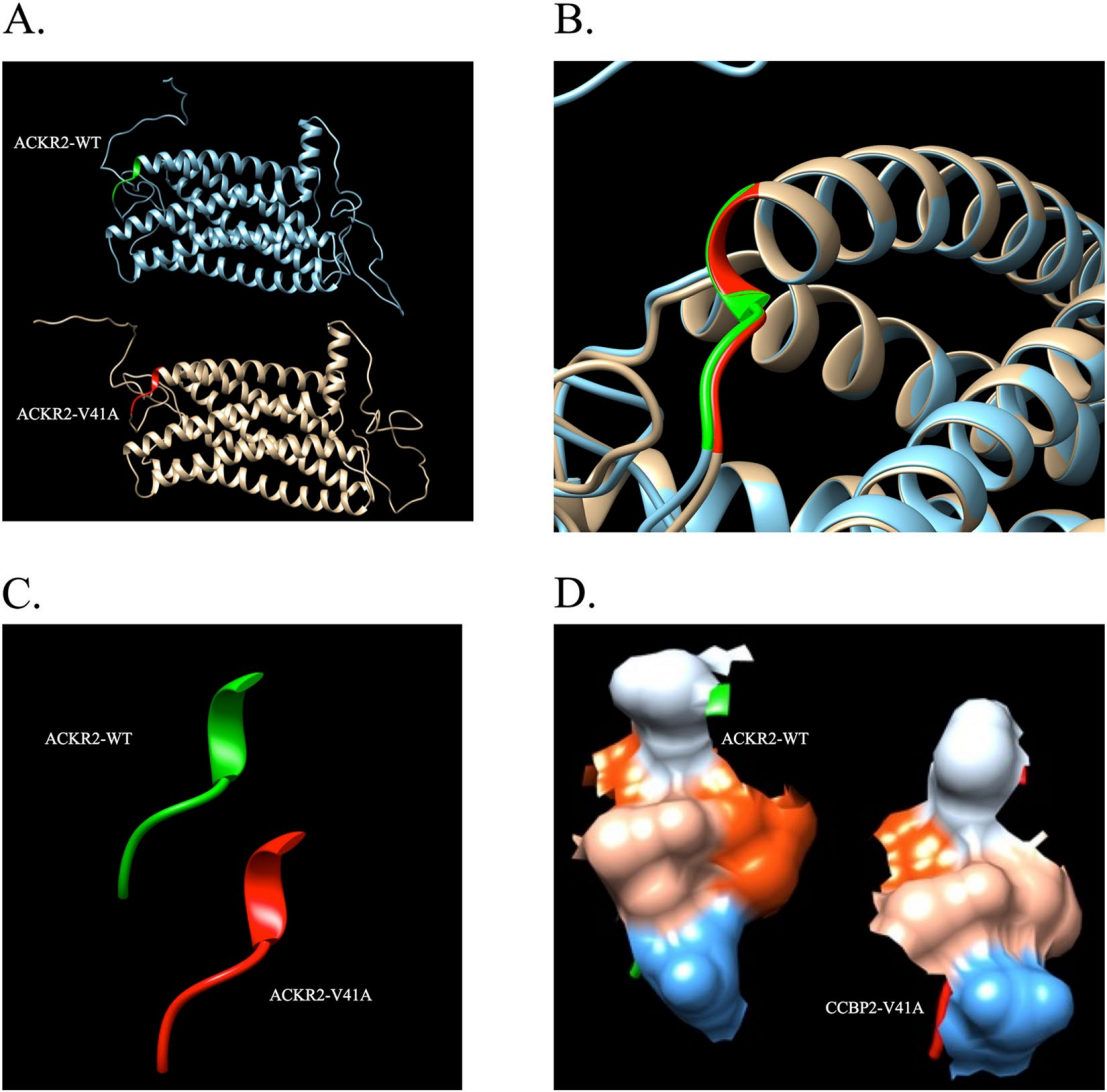
Model of ACKR2-V41A does not predict a change in protein folding and predicts a minor decrease in hydrophilicity. There is no dramatic difference in predicted protein folding and a minor decrease in hydrophilicity between ACKR2-WT and ACKR2-V41A. (**A**) Side by side ribbon view of ACKR2-WT (blue and green) and ACKR2-V41A (gold and red) protein models. (**B**) Overlay of ACKR2-WT and ACKR2-V41A ribbon models highlighting the folding at amino acid position 41 (WT is green and V41A is red). (**C**) Side by side ribbon structure of amino acid position 39–43 for ACKR2-WT and ACKR2-V41A (WT is green and V41A is red) (**D**) View of hydrophobic (red) and hydrophilic (blue) amino acids at amino acid positions 39–43 for the ACKR2-WT (left) and ACKR2-V41A (right) chemokine binding site. Amino acid 41 is shown in the middle right of both models.

ACKR2-V41A differs from wild type by a single amino acid located at the chemokine binding site of ACKR2. V41A is specifically located in the first of three extracellular loops. Predicted protein folding models were generated and ACKR2-WT and ACKR2-V41A were superimposed to check for differences in folding and hydrophilicity. There was no observed difference in predicted protein structure folding (Fig. 1A,B).

### ACKR2-V41A is predicted to have minor hydrophilicity changes compared to wild type

The single amino acid change at position 41 is predicted to have a slight decreased hydrophilicity between ACKR2-WT and ACKR2-V41A (Fig. 1C,D). Despite a lack of predicted folding and a minor hydrophilicity difference, this variant was predicted to have a biological effect. Annotation analysis with *RegulomeDB* gave this nonsynonymous mutation a score of 5/6. *PolyPhen-2* predicted this variant to be probably damaging with a score of 0.958 (sensitivity: 0.78; specificity: 0.95). These predictor tools - Chimera, RegulomeDB and PolyPhen-2 all predicted that the ACKR2-V41A variant causes a biological change with functional consequences.

### ACKR2-V41A and ACKR2-WT have similar receptor expression levels

**Figure 2:**
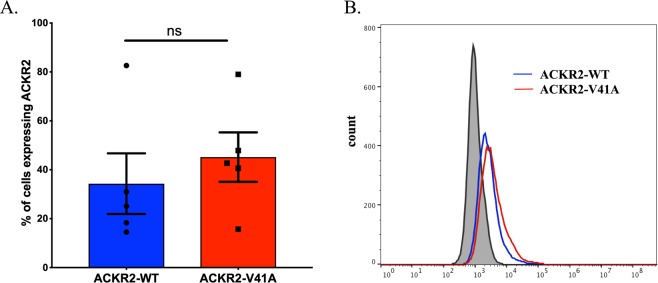
Atypical chemokine receptor ACKR2-V41A and ACKR2-WT have similar levels of receptor expression on the cell surface. (**A**) There is no significant difference in the percent of positive cell for the atypical chemokine receptors ACKR2-V41A and ACKR2-WT (n = 5; ns = not significant) at the cell surface. (**B**) ACKR2-V41A and ACKR2-WT have similar cell expression levels (n = 3).

CHO-k1 cells were transfected with ACKR2-WT or ACKR2-V41A and were grown in selective media for a week before transfection positive cells were enriched using beads and magnetic columns. The enriched CHO-k1 ACKR2-WT or ACKR2-V41A cell populations had similar percentages of receptor positive cells (Fig. 2A) and receptor expression levels (Fig. 2B). T-test analysis (n = 5) failed to detect a significant difference between the percentage of cells expressing ACKR2 WT or ACKR2-V41A at the cell surface. Indicating the transfection efficiency between cell populations ACKR2-WT and ACKR2-V41A are not different.

### ACKR2-V41A has decreased binding affinity for CCL2 compared to ACKR2-WT

**Figure 3:**
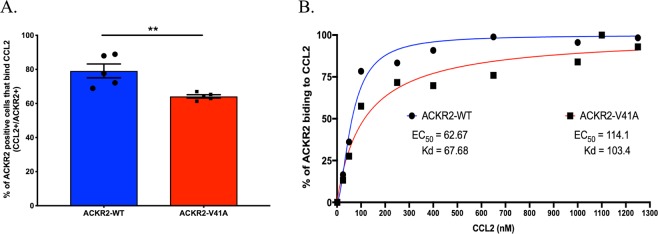
CCL2 binds with lower affinity to ACKR2-V41A compared to ACKR2-WT in ligand binding assay. (**A**) ACKR2-WT and ACKR2-V41A transfected cells were incubated with anti-ACKR2 antibody and a separate tube of ACKR2-WT and ACKR2-V41A transfected cells were incubated with AF647-conjugated CCL2. After normalizing the percentage of CCL2 + cells over ACKR2 + cells, there is a significantly lower binding affinity between ACKR2-V41A than ACKR2-WT for CCL2 (two tailed T test; p-value 0.0071) (n = 5; **p < 0.01). (**B**) The same experiment was repeated using different concentrations of AF647-conjugated CCL2 ligand (ranging from 50 nM to 1200 nM). ACKR2-WT was saturated when 650 nM of CCL2 ligand was used (n = 3), yielding a 1.5 lower Kd value (67.68 nM) than ACKR2-V41A (103.4 nM).

The binding affinity of ACKR2-WT and ACKR2-V41A for CCL2 was measured in both a direct binding and a competition assay. In the direct binding assay, the percentage of CCL2 binding to ACKR2-WT and ACKR2-V41A was determined by measuring the levels of AF647-conjugated CCL2 divided by the ACKR2 receptor levels. Analysis of 5 independent runs revealed that ACKR2-V41A has a significantly lower binding affinity than ACKR2-WT (Fig. 3A; two tailed T test, p-value 0.0071). The ratio between percentage of CCL2 bound to the receptor over percentage of chemokine receptor expression is lower in cells with ACKR2-V41A (μ = 64.16) than cells expressing ACKR2-WT (μ = 79.12). These affinity differences were consistent in 3 independent runs using a titration of AF647-conjugated chemokine ligand CCL2 starting at 50 nM and reaching saturation for ACKR2-WT and V41A variant at 1200 nM (Fig. 3B). Non-linear analysis showed that ACKR2-V41A has a 1.5-fold higher equilibrium dissociation constant (Kd = 103.4 nM) than ACKR2-WT (Kd = 67.68 nM) (Fig. 3B). A similar method was followed to observe if AF647-conjugated CCL19 was capable of interacting with either ACKR2-WT or ACKR2-V41A; however, there was no detectable interaction (Supplemental Fig. 1)

A competition assay was also performed to test if the unconjugated chemokine ligand CCL2 was able to interfere with binding of an anti-ACKR2 antibody. Analysis of 5 independent runs showed a significant difference between chemokine receptor ACKR2-WT and ACKR2-V41A (two-tailed T test, p-value 0.0153). ACKR2-V41A expressing cells bound to significantly lower levels of CCL2, allowing the ACKR2 antibody to bind at a higher percentage (μ=88.5) than ACKR2-WT (μ = 66.18). Quantification of levels of unconjugated CCL2 blocking the anti-ACKR2 antibody show that ACKR2-V41A (11.5%) binds with significantly lower affinity to CCL2 than ACKR2-WT (33.82%) (Supplemental Fig. 2).

### ACKR2-V41A has decreased binding affinity for CCL4 compared to ACKR2-WT

**Figure 4:**
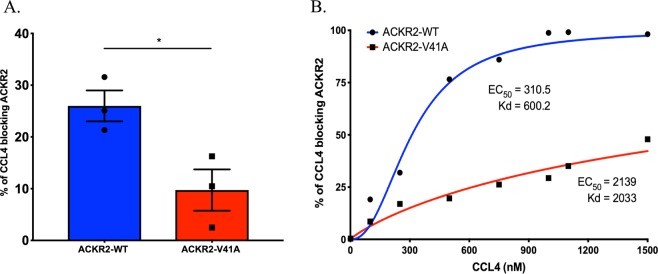
CCL4 binds with lower affinity to ACKR2-V41A compared to ACKR2-WT in ligand binding assay. (**A**) Cells were incubated with unconjugated CCL4 for 45 minutes at 37 °C; then, anti-ACKR2 was added to determine levels of receptor binding inhibition by CCL4. ACKR2 bound to CCL4 with lower affinity, resulting in higher ACKR2 receptor binding with the anti-ACKR2 antibody compared to ACKR2-WT. 100 minus the percentage of ACKR2 + cells were subtracted to determine the percentage of CCL4 blockage. ACKR2-V41A (9.74%) was blocked at a significantly lower percentage by CCL4 than ACKR2-WT (26.0%) (n = 5; *p < 0.05). (**B**) The same experiment was repeated using different concentrations of CCL4 ligand (ranging from 100 nM to 1200 nM). ACKR2-WT was saturated at 1000 nM of CCL4 ligand (n = 3) yielding a 3.4-fold lower Kd value (600.2 nM) than ACKR2-V41A (2033 nM).

The binding affinity of ACKR2-WT and ACKR2-V41A for CCL4 was measured in a competition assay using an unconjugated CCL4 ligand to block the ACKR2 antibody. This competition analysis aligned with the CCL2 binding results with CCL4 blocked at a statistically lower percentage by ACKR2-V41A (9.74%) than ACKR2-WT (26.0%) (Fig. 4A; two-tail T test, p-value 0.0310). These binding affinity results were consistent in 3 independent runs using a titration of unconjugated CCL4 starting at 100 nM and reaching saturation for ACKR2-WT at 1000 nM (Fig. 4B). Under this circumstances, ACKR2-V41A did not reach full saturation. Non-linear analysis showed that ACKR2-V41A has a 3.4-fold higher Kd value (2033 nM) than ACKR2-WT (600.2 nM).

### ACKR2-V41A has decreased CCL2 scavenging efficiency compared to wild type

**Figure 5:**
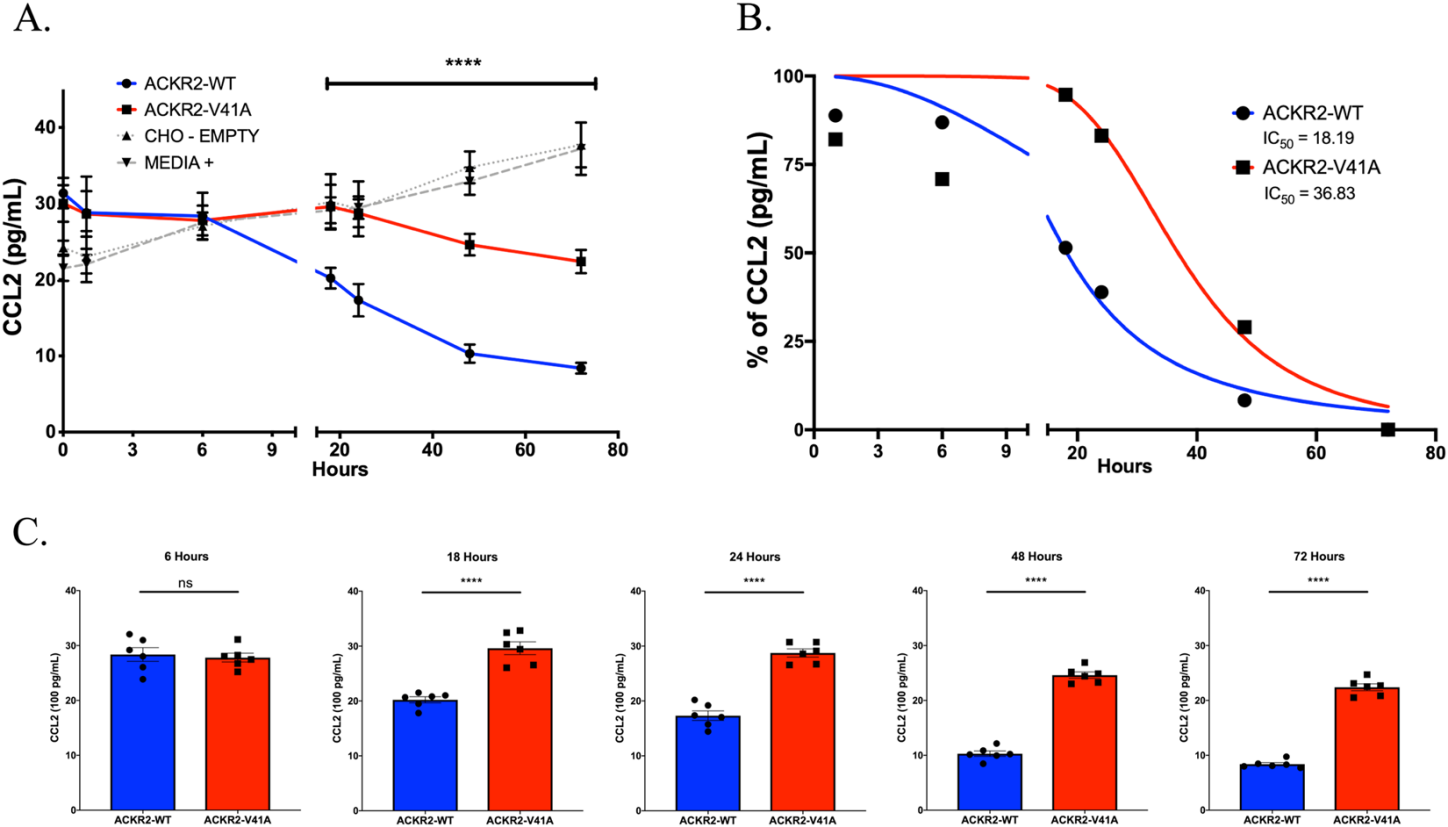
ACKR2-V41A has a significantly lower scavenging efficiency for CCL2. (**A**) CCL2 scavenging efficiency measurements. CHO-k1 cells expressing ACKR2-WT and ACKR2-V41A were incubated with an initial concentration of 100 pg/mL of CCL2. CCL2 levels were measured over the course of 72 hours. Cells with atypical chemokine ACKR2-V41A scavenged significantly lower levels of CCL2 compared to ACKR2-WT (two tailed T test, p-value 0.0097) (n = 5; ****p < 0.0001). (**B**) ACKR2-V41A has a two-fold higher IC_50_ value (36.83 hours) than ACKR2-WT (18.19 hours). (**C**) Scavenging analysis by time point. At 6 hours ACKR2-WT and ACKR2-V41A were scavenging at the same rate. After 18 hours, cells with receptor ACKR2-V41A scavenged CCL2 ligand at a significantly lower rate and this continued up to 72 hours (n = 5; ****p < 0.0001).

One of the main roles of the atypical chemokine receptor ACKR2 is to scavenge CCL2. To determine scavenging efficiency, we incubated ACKR2-WT and ACKR2-V41A expressing cells with CCL2 and measured levels of CCL2 in the supernatant over the course of 72 hours. ACKR2-V41A cells scavenged CCL2 at a significantly lower efficiency than cells expressing ACKR2-WT (Fig. 5A; two tailed T test, p-value 0.0097). There is a significant difference in scavenging efficiency between ACKR2-WT and ACKR2-V41A starting at the 18 hour time point until the last measurement at 72 hours (Fig. 5B,C; two tailed T test, p-value <0.0001 at 18, 24, 48, and 72 hours). ACKR2-WT has an IC_50_ of 18.19 hours, illustrating that the WT receptor scavenges CCL2 more efficiently than ACKR2-V41A, which has an IC_50_ of 36.83 hours (Fig. 5B; non-linear regression analysis). The increase in CCL2 levels in the CHO-empty and media wells is likely due to evaporation over the course of 80 hours.

### ACKR2-V41A has decreased receptor upregulation compared to wild type

**Figure 6:**
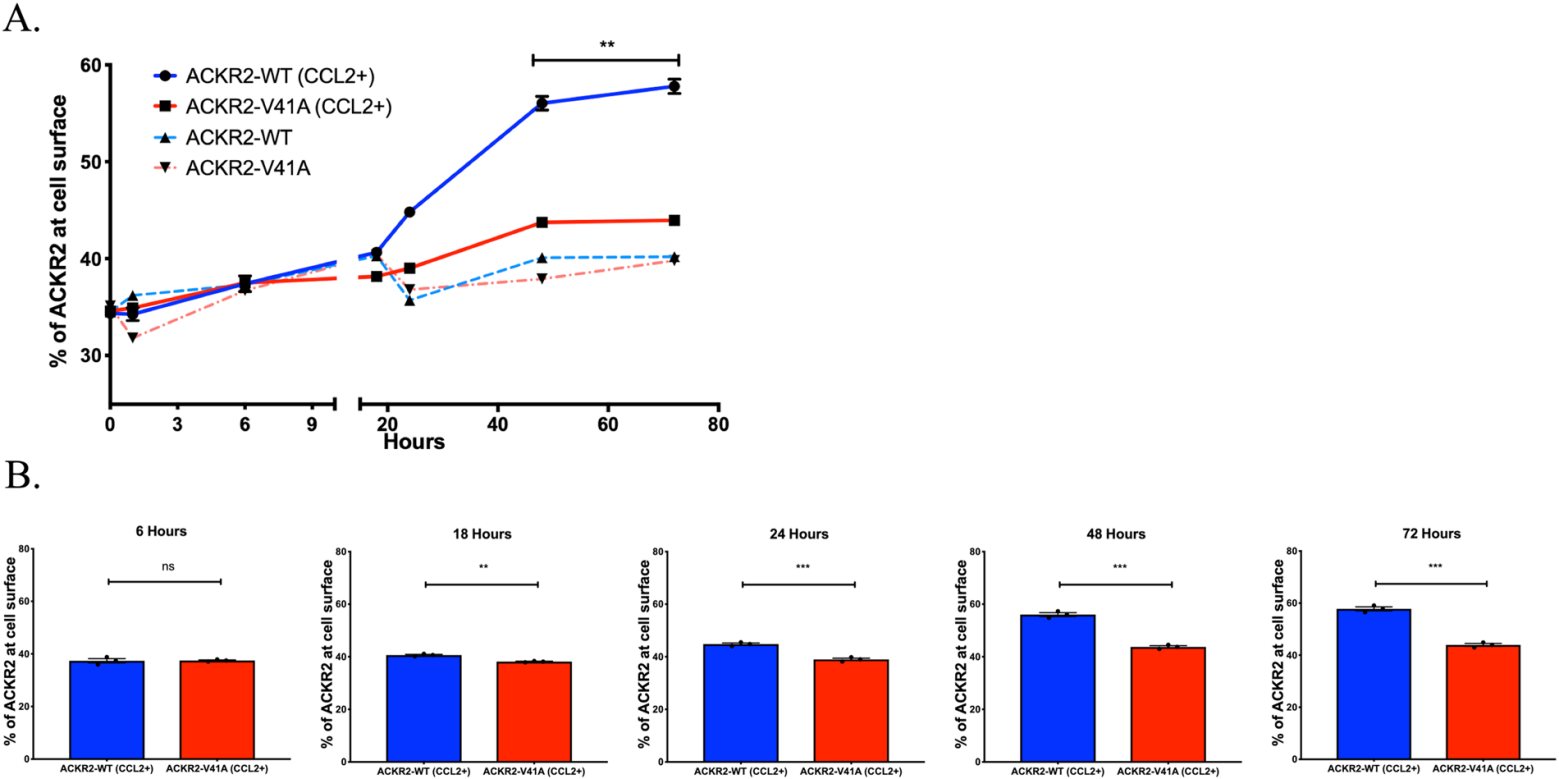
ACKR2-V41A has significantly lower receptor expression upon CCL2 stimulus. (**A**) ACKR2-V41A and WT receptor expression over 72 hours. CHO-k1 cells expressing ACKR2-WT and ACKR2-V41A were incubated with an initial concentration of 100 pg/mL of CCL2. Receptor expression was measured over the course of 72 hours. Cells with atypical chemokine ACKR2-V41A responded significantly lower to the stimulus of CCL2 compared to ACKR2-WT (two tailed T test, p-value 0.0045) (n = 3; **p < 0.01). (**B**) Analysis of receptor levels by time points. At the six hour mark ACKR2-WT and ACKR2-V41A were expressing the similar amount of receptor at the cell surface. After 18 hours, cells with receptor ACKR2-V41A were expressed at a significantly lower percentage and this continued up to 72 hours (n = 3; **p < 0.01, ***p < 0.001).

An increase in receptor recycling and ACKR2 receptor expression levels on the cell surface is stimulated by the presence of a chemokine ligand capable of activating the receptor^55^. ACKR2-WT and ACKR2-V41A cell surface receptor levels were monitored for 72 hours after the CCL2 chemokine was added to the supernatant. In this assay, cells expressing ACKR-WT or ACKR2-V41A started with similar expression levels and maintained similar levels for 6 hours, but from the 18 hour to the 72 hour time points ACKR2-V41A had significantly lower levels of receptor expression (Fig. 6A; two tailed T test, p-value 0.0045 and Fig. 6B; two tailed T test, p-value <0.01)

### ACRK2-V41A has a lower expression of phosphorylated cofilin compared to wild type

**Figure 7:**
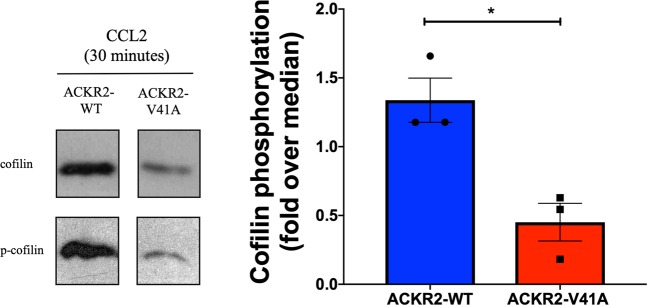
ACKR2-V41A has lower levels of phosphorylated cofilin after activation with CCL2 ligand. The G protein-dependent response of CHO-k1 cells with atypical chemokine ACKR2-WT and ACKR2-V41A receptors were measured by presence of p-cofilin in either receptor. P-cofilin levels were measured after activation with CCL2 for 30 minutes. Representiative western blots are shown on the left and data analysis of three runs are shown on the right. Data was normalized using cofilin. Cells expressing ACKR2-V41A had significantly lower p-cofilin than cells expressing ACKR2-WT (n = 3, two tail T test, p-value 0.0137).

To confirm that the ACKR2 receptor upregulation differences were dependent on the β-Arrestin-dependent signaling pathway, we measured cofilin phosphorylation. Cofilin is part of the β-Arrestin-dependent signaling pathway and is critical to the regulation of chemokine activation, recycling, and production of the ACKR2 receptor. CHO-k1 cells expressing either ACKR2-WT or ACKR2-V41A receptor were examined via western blot to determine the levels of phosphorylated cofilin (p-cofilin) induced by CCL2. Upon activation of the receptors with chemokine ligand CCL2, cells expressing ACKR2-V41A receptor had significantly lower p-cofilin levels than cells expressing ACKR2-WT (Fig. 7 and Supplemental Fig. 4; two tailed T test, p-value 0.0137).

### ACKR2-V41A has decreased activation sensitivity compared to wild type

CHO-k1 cells expressing either ACKR2-WT or the ACKR2-V41A chemokine receptor activation profiles were also examined using live-cell calcium (Ca^2+^) imaging. Upon activating the receptor with chemokine CCL2, cells expressing the ACKR2-V41A receptor have significantly lower levels of calcium signaling starting 4 minutes after CCL2 activation when compared to CCBP-WT (Supplemental Fig. 3A; two tailed T test, p-value 0.0052). Analysis of the area under the curve from minute 1 (addition of CCL2) to minute 5 (end time point) showed that ACKR2-V41A cells have significantly lower levels of calcium signaling after CCL2 activation compared to ACKR2-WT (Supplemental Fig. 3B; two tailed T test, p-value 0.008).

## Discussion

We have identified direct biological effects of the ACKR2-V41A variant (rs2228467) and a possible mechanism for increased levels of CCL2 in the CSF. ACKR2-V41A has lower CCL2 binding affinity, CCL2 scavenging efficiency, and CCL2 induced ACKR2 receptor upregulation. As proposed in the original GWAS analysis^20^, increased CSF CCL2 levels may be due to decreased binding affinity of ACKR2-V41A for CCL2. We also found that the ACKR2-V41A variant also has altered binding to the chemokine CCL4 and an important area to address in future studies will be how its binding is altered to all 12 of its known ligands.

Chimera protein predictor software, PolyPhen-2, and Regulomedb predicted that the amino acid change from the hydrophobic valine to the less hydrophobic alanine was sufficient to change biological function. In this study, we demonstrated that variant V41A, located at the first extracellular loop and binding site, reduces the binding affinity of receptor ACKR2 with CCL2 and CCL4 ligands. We suspect that same behavior will be observed if the binding affinity of this variant is tested with the remaining 12 chemokine ligands known to interact with it. In addition, ACKR2-WT and ACKR2-V41A did not noticeably improve binding for the chemokine ligand CCL19. This ligand is one of the chemokines from the CC family that does not interact with ACKR2 receptor. Thus, in this case the V41A variant did not enable the ACKR2 receptor to interact with a new chemokine ligand.

This altered ability of ACKR2 to bind CCL2 resulted in decreased CCL2 scavenging activity and lower levels of receptor on the cell surface. ACKR2-V41A lost its scavenging efficiency over time (after 18 hours of saturating the system with 100 pg/ml of CCL2) and also has decreased levels of the ACKR2 receptor at the cell surface over time (starting at 18 hours after the cells were stimulated with 100 pg/ml of CCL2) compared to wild type. We found that ACKR2-V41A is two-fold less efficient at scavenging CCL2, resulting in the maintenance of this inflammatory chemokine signal for a longer time than wild type. These results suggest that binding affinity and receptor levels are key to regulating levels of pro-inflammatory chemokines such as CCL2.

ACKR2 is an atypical chemokine receptor and lacks the signaling motif to initiate the production of other chemokine and cell migration. ACKR2 stimulation by CCL2 activates the β-Arrestin-dependent signaling pathway cofilin pathway [Rac1-p21-activated kinase 1 (PACK1)-LIM kinase 1 (LIMK1) cascade] and ACKR2 can send an intracellular signal to upregulate surface receptor levels^38,54,55^. Here we report that after CCL2 activation, p-cofilin expression levels were reduced in cells with the ACKR2-V41A receptor compared to wild type. This result aligns with the assay examining ACKR2 receptor levels after CCL2 stimulation over a 72 hours period. Low levels of p-cofilin in ACKR2-V41A cells could explain the low level of ACKR2 quantified at the cell surface and the decreased CCL2 scavenging activity. In addition, there are compelling studies showing that ACKR2 is capable of Ca^2+^ mobilization upon stimulation with CCL2 in in HEK 293 cells^56^ and neurons^23,57^. These studies align with our results and strengthen our proposed mechanism. Here we have shown a reduction of the biological capacity of the atypical chemokine ACKR2-V41A to bind, scavenge, and be activated by CCL2. Additional impacts of the ACKR2-V41A allele should also be examined in regard to the other 12 chemokines ACKR2 is known to bind with^37^. Thus, alterations in chemokine levels caused by ACKR2-V41A have a powerful effect on chemokine gradient homeostasis, inflammation, and increased risk to develop AD.

In the presence of Aβ aggregation the expression and production of pro-inflammatory related molecules, including CCL2, are elevated. In this context, it is known that high levels of CCL2 expression and production trigger microgliosis, increase the APP/CCL2 ratio, and increase Aβ plaque aggregation. CCL2 could increase the rate of Aβ deposition by interference with clearance as well as increase expression of apolipoprotein E, a main biomarker for AD pathogenesis and Aβ deposition^30,58,59^. Thus, regulation of CCL2 levels has multiple effects on brain homeostasis and longevity. In this study, we observed significant differences in the ability of ACKR2-V41A to bind and scavenge CCL2, potentially resulting in higher levels of CCL2 at the injury site and maintenance of pro-inflammatory signaling and monocyte recruitment, which may lead to increased Aβ plaque aggregation and risk for AD.

GWAS researchers have identified thousands of genetic variants contributing to the rate of development of complex human diseases; providing insights at the DNA level of how a single nucleotide polymorphism (SNP) might cause changes in function at the biological and molecular level. The characterization of biological mechanisms by which rare variants found in GWAS to modulate risk for disease has proven to be extremely difficult. SNP characterization requires a deeper understanding of how DNA changes might affect gene expression through transcription, RNA splicing, ligand-receptor binding, protein function etc^60^. Consequently, genetic variants identified by GWAS have to be physiologically significant to conclusively identify their biological effects as proteins. In this study we provide the biological characterization of ACKR2-V41A that was identified using a GWAS. We have provided insights into the mechanism by which ACKR2-V41A with CCL2 alters the chemokine gradient homeostasis, increases inflammation, and the alters risk for AD. Future efforts should focus understanding if and how ACKR2-V41A impacts Aβ production and aggregation and other biomarkers of AD in AD-relevant cell lines.

## Methods

### Chimera

ACKR2-WT and ACKR2-V41A chemokine receptor amino acids sequences were sent in a FASTA format to the University of Michigan’s Zhang Lab LOMETS server. 3D models were generated by collecting high-scoring structural templates from 11 locally-installed threading programs (CEthreader, FFAS3D, HHpred, HHsearch, MUSTER, Neff-MUSTER, PPAS, PRC, PROSPECT2, SP3, and SparksX)^61,62^. These 11 models were compared to similar chemokine receptors for accuracy. Using UCSF Chimera Software, the 3D models were oriented in space^63^. We superimposed ACKR2-WT and ACKR2-V41A to check for differences in folding and hydrophilicity. 3D images were produced using UCSF Chimera Software to highlight the predicted chemokine receptor structure and hydrophobic interactions at amino acid position 41^63^.

### Annotation

Annotation and prediction of the biological function of ACKR2-V41A variant was determined using RegulomeDB and Polymorphims Phennotyping v2 (PolyPhen-2). RegulomeDB is a database that annotates variants with known and predicted regulatory elements in human genome such as eQTL, transcription factor binding and transcription factor motif^64^. This predicter algorithm scores between 1 and 6 and any variant categorized between 1 and 5 is predicted to have damaging biological function in the human genome. PolyPhen-2 is an algorithm that predicts possible biological impact of an amino acid substitution in the human genome^65^. This algorithm classifies the biological impact of a variant from a damaging to a tolerable variant.

### Transformation and transfection

Plasmid construct were ordered at Genscript. DNA sequences for ACKR2-WT and ACKR2-V41A were inserted into vector pCMV6-AN-myc-DDK (ORIGENE, Cat #PS100016). Transformation protocols were followed as recommended by the manufacturer. Briefly, plasmids were transformed into DH5*a* cells, amplified, and purified using the ZymoPURE II Plasmid Maxiprep kit (Cat #D4203). Plasmids were transfected into Chinese Hamster Ovary (CHO-k1 ATCC® CCL-61) cells using lipofectamine (ThermoFisher, Cat #15338100) and manufacture recommendations were followed. Properly transfected cells were selected using the antibiotic G418 sulfate (ThermoFisher, Cat #10131035). Transfected cells were grown in F12 media (10 μg/ml penicillin, 10 μg/ml streptomycin; Gibco Cat #21127-022) with 10% FBS (HYCLone Cat #SH30071.01). Cell media was changed every 48 to 72 hours depending on cell confluency levels.

### Cell isolation

We tested the atypical chemokine receptor ACKR2-V41A function using CHO-k1 cells because they lack expression of the wild type ACKR2 receptor as well as other competing human chemokine receptors. It is important to note that this cell type is absent in the human brain and therefore our results should not be overinterpreted. After transfecting CHO-k1 cells with plasmids carrying ACKR2-WT or ACKR2-V41A, cells were incubated until they reached 80% confluency and prepared for cell sorting. Cells were stained for 30 minutes at 37 °C with anti-human ACKR2 polyclonal IgG antibodies (ABNOVA Cat #H00001238-B02). After two washes, cells were stained for 30 minutes at 4 °C with anti-human IgG conjugated with PE (Biolegend Cat #405307). Cells were then washed and isolated using anti-PE MicroBeads from MACS Miltenyi Biotech (Cat #130-048-801) following manufacture recommendations. After isolation, cells were incubated in F12 media until 80% confluency was reached.

### Receptor expression

CHO-k1 cells transfected with ACKR2-WT or ACKR2-V41A were harvested when a 75 cm^2^ flask reached 80% cell confluency. 250,000 cells were resuspended in 300 μL of PBS-1% BSA and placed in FACs tubes. To evaluate expression of the ACKR2 receptor, cells were stained with anti-CD16/32 (Fc block) on ice for 20 minutes (eBioscience, Cat #14-0161-85). After two washes, cells were stained for 30 minutes at 37 °C with anti-human ACKR2 polyclonal IgG antibodies, washed twice, and then stained for 30 minutes at 4 °C with anti-human IgG conjugated with PE. After two washes receptor expression was analyzed on a BD Accuri. In addition, expression of the receptor at cell surface was monitor for 72 hours after CCL2 was added to the growing media. Expression levels were obtained following the procedures above.

### Receptor binding

To determine CCL2 binding levels for ACKR2-WT and ACKR2-V41A, cells were incubated with conjugated CCL2-AF647 (ALMAC, Cat# CAF-02-D-2) at 37 °C for 45 minutes at different concentrations ranging between 100 to 750 nM. After two washes receptor expression was analyzed on a BD Accuri. To determine the levels of ACKR2-WT and ACKR2-V41A receptor expression, cells were stained with anti-CD16/32 (Fc block) on ice for 20 minutes. After two washes, cells were stained for 30 minutes at 37 °C with anti-human ACKR2 polyclonal IgG antibodies, washed twice, and then stained for 30 minutes at 4 °C with anti-human IgG conjugated with PE. In the CCL2 competitive binding assay levels for ACKR2-WT and ACKR2-V41A, cells were incubated with unconjugated CCL2 (PEPROTECH, Cat #300-04100UG) at 37 °C for 45 minutes. After two washes and an Fc block as described above, cells were stained for 30 minutes at 37 °C with anti-human ACKR2 polyclonal IgG antibodies, washed twice, and then stained for 30 minutes at 4 °C with anti-human IgG conjugated with PE. After two washes, levels of binding of the ACKR2 antibody was analyzed in BD Accuri.

CCL4 (PEPROTECH, Cat #300-.9) competition binding assay was performed following the procedures above. Conjugated CCL19-AF647 (ALMAC, Cat #CAF-06-A-01) binding analyses were completed using the same methods used for CCL2 binding assay.

### ELISA

ACKR2-WT and ACKR2-V41A cells were seeded in a 6-well cell culture plate with fresh F12 media with 10% FBS and 100 pg/mL of CCL2 added to each well. At varying time points, 150 μL of media supernatant was extracted from each well to measure the remaining concentration of CCL2 using a CCL2 TMP sandwich ELISA kit (PeproTech, Cat #900-T31). Ninty-six well ELISA microplates were coated overnight at room temperature with a monoclonal antibody specific for human CCL2 (0.25 μg/mL in PBS). Plates were washed three time with 0.05% tween diluted in PBS and then wells incubated with blocking buffer (PBS/1% BSA) for 2 hours at room temperature. After three washes, samples and controls were added to the wells and incubated for 2 hours at room temperature. After three washes plates were incubated for 1 hour at room temperature with biotinylated anti-human CCL2 antibody (0.25 μg/mL) diluted in blocking buffer. After three washes, plates were incubated with 100 μL of Streptavidin-HRP conjugate (0.05 μg/mL) for 30 minutes at room temperature. Plates were washed three times and 100 μL of TMB substrate solution (hydroperoxide and chromogen tetramethylbenzidine) was added for 20 minutes at room temperature in the dark. The reaction was stopped with 100 μL of 1 M of hydrochloric acid. Optical density was determined using a micro plate reader set at 450 nm with a wavelength correction of 620 nm.

### ACKR2 activation pathway

Levels of phosphorylated cofilin were obtained following methodology from previously described research^55^. Briefly, 300,000 CHO-k1 cells expressing either ACKR2-WT or ACKR2-V41A were seeded in a well and serum-starved for 18 hours. Then, cells were stimulated with 100 nM of CCL2 (PEPROTECH, Cat #300-04100UG) and incubated at 37 °C in 5% CO_2_ for 30 minutes. Cells were harvest, lysed using RIPA lysing buffer (ThermoFisher, Cat #89900) following manufacture recommendations. Lysed product was prepared for a western blot assay. Two gels were run in parallel using the same lysed product samples. One gel was stained with antibody for p-cofilin (Cells signaling, Cat #3313) to detect phosphorylated cofilin. ImageJ (ImageJ software, NIH) was used to analyze the intensity of bands corresponding to p-cofilin. The second gel was stained with cofilin (Cell signaling, Cat #5175) and was used for normalization of p-cofilin after ImageJ analysis. An independent T test was performed to determine if p-cofilin levels differ between cell expressing ACKR2-WT and ACKR2-V41A.

### Calcium activation

Cells were seeded on a 8-chambered slide (Nunc, Cat #155411) and incubated overnight in F12 media with 10% FBS at 37 °C in 5% CO_2_. The next day, cells were loaded with 4 μM of Fura-2AM (Invitrogen, Cat #F1221) in Ringers solution (150 mM NaCl, 10 mM glucose, 5 mM HEPES, 5 mM KCl, 1 mM MgCl_2_, 2 mM CaCl_2_, pH 7.4) for thirty minutes at 37 °C in 5% CO_2_. Cells were then washed once with Ringers solution and incubated for another 30 minutes at 37 °C in Ringers solution. Calcium imaging was performed at room temperature using an Olympus IX51 inverted microscope equipped with a xenon arc lamp. Fura-2AM loaded CHO-k1 cells expressing receptor ACKR2-WT and ACKR2-V41A were excited using 340 nm and 380 nm excitation filters, and images of 340 nm, 380 nm, and transmitted light was captured using a fluorescence microscope camera (Q Imaging Exi Blue) with a 20x objective (N.A. 0.75) at 1.6-sec intervals. At the one minute time point in each imaging protocol, 250 nM of recombinant Human CCL2 was added to stimulate Ca^2+^ flux^23^. Ionomycin (1 μM final concentration) was added at the five minute time point as a positive control. Ten to twenty representative cells were selected as regions of interest in each frame, and F340:F380 ratios were calculated and analyzed using CellSens software from Olympus. Each individual cell’s fluorescence was normalized to its first recorded value according to the equation (F-Fo)/Fo, where F is the fluorescence at the specific time point, and Fo is the fluorescence value at time 0^66,67^.

### Statistical analysis

Statistical analyses were performed using the Prism 8 software (GraphPad Software, La Jolla, CA, USA). Differences in binding affinity, CCL2 scavenging efficiency, and cell activation sensitivity between ACKR2-V41A and ACKR2-WT were analyzed using an unpaired T-test. Kd, EC_50_, and IC_50_ were calculated using non-linear analysis best fit test. Each independent run contained a minimum of three replicates per sample and the mean of those replicates were considered a single point of analysis. In this study, each experiment was performed with three to five independent runs (9 to 15 replicates per sample). Results presented are representative of observed phenotypes and analysis with P-values less than 0.05 were considered significant.

## Supplementary information

Supplementary information.
