# Supplementary material for: Atypical chemokine receptor ACKR2-V41A has decreased CCL2 binding, scavenging, and activation, supporting sustained inflammation and increased Alzheimer’s disease risk
Source: Sci Rep. 2020 May 15;10:8019. doi: 10.1038/s41598-020-64755-1 (PMC7229167; doi:10.1038/s41598-020-64755-1)
Supplement: Supplementary file 1 — Supplementary information. [file 41598_2020_64755_MOESM1_ESM.pdf]

Supplemental Material

Atypical chemokine receptor ACKR2-V41A has decreased CCL2 binding, scavenging, and activation, supporting sustained inflammation and increased Alzheimer's disease risk

Josue D. Gonzalez Murcia[1], Allen Weinert[2], Claudia M. Tellez Freitas[2,3], Daniel K. Arens[2], Meganne Ferrel[1], Julianne H. Grose[2], Perry G. Ridge[1], Eric Wilson[2], John S. K. Kauwe[1*], K. Scott Weber[2*]

[1] Brigham Young University
Department of Biology
4102 Life Sciences Building
Provo, Utah 84602

[2] Brigham Young University
Department of Microbiology & Molecular Biology
4007 Life Sciences Building
Provo, Utah 84602

[3] Roseman University of Health Science
College of Dental Medicine
10894 S. River Front Parkway
South Jordan, UT 84095

* Co-senior authors

Corresponding authors:

K. Scott Weber (scott_weber@byu.edu) or John S. K. Kauwe (kauwe@byu.edu)

Supplemental figure 1

A.

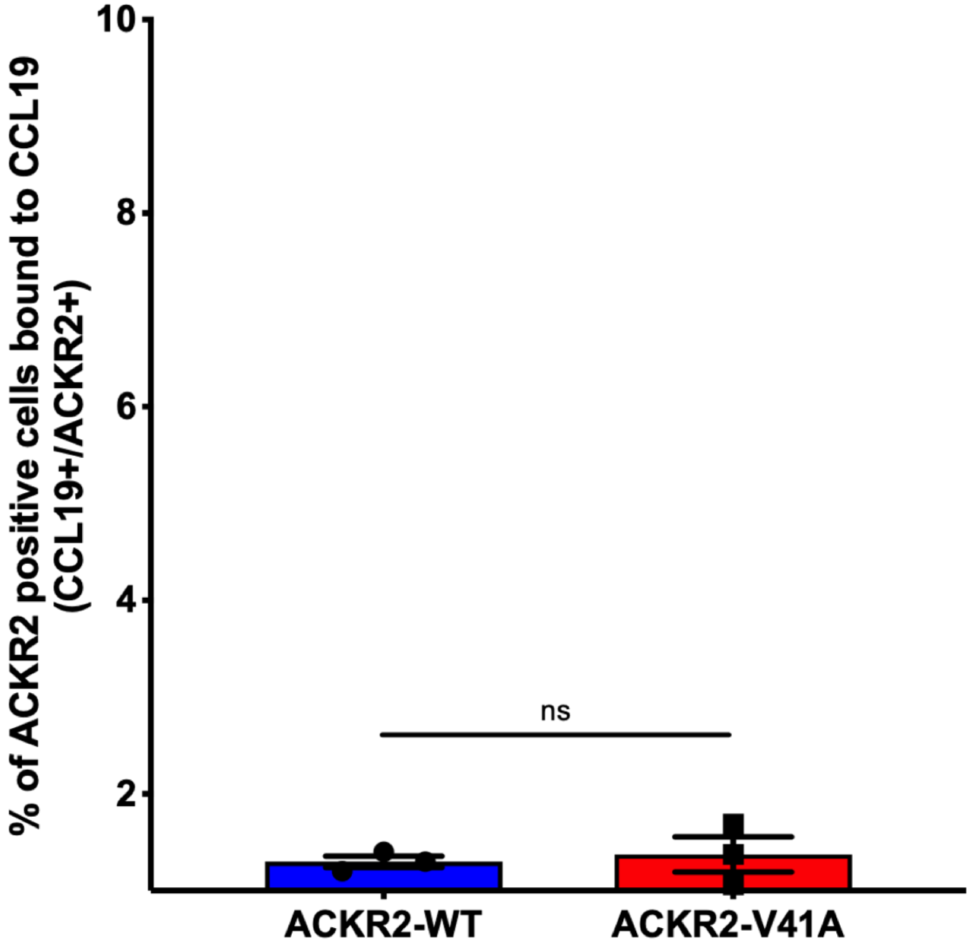

Supplemental figure 1. **CCL19 does not interact with ACKR2 receptor.** Atypical chemokine receptors ACKR2-WT and ACKR2-V41A were compared in a binding affinity assay with CCL19. ACKR2-WT and ACKR2-V41A transfected cells were incubated with conjugated anti-ACKR2 antibody and a separate tube of ACKR2-WT and ACKR2-V41A transfected cells were incubated with conjugated CCL19. After normalizing the percentage of CCL19+ cells over ACKR2+ cells, we failed to detect interaction between CCL19 with ACKR2-WT and ACKR2-V41A.

Supplemental figure 2

A.

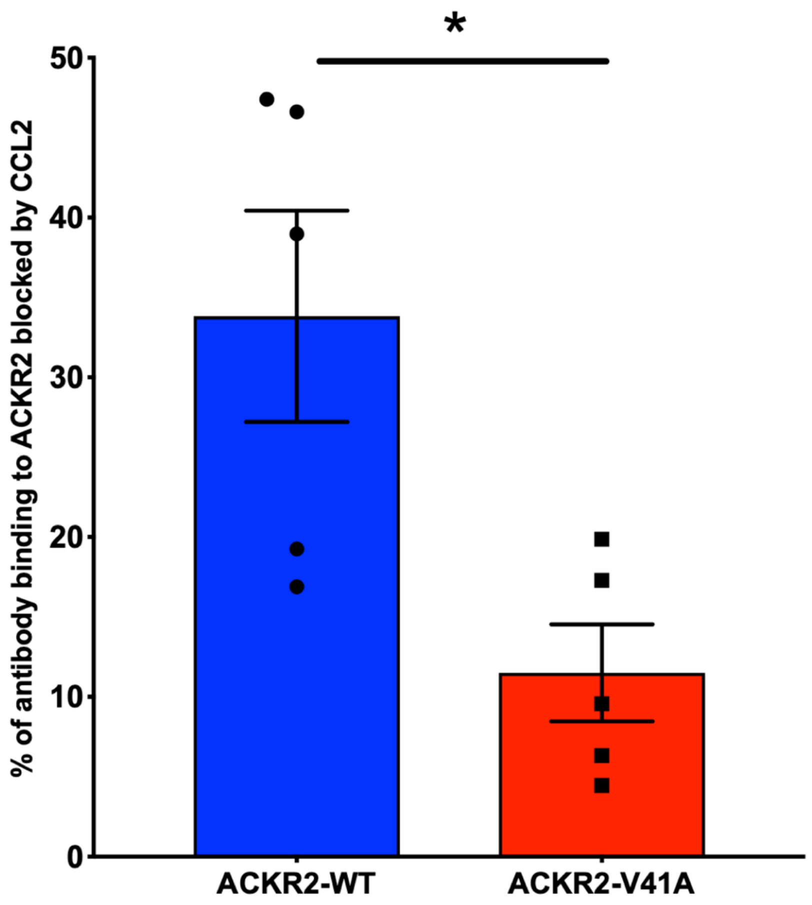

Supplemental figure 2. **CCL2 binds with lower affinity to ACKR2-V41A in a competition binding assay.** Atypical chemokine receptors ACKR2-WT and ACKR2-V41A were compared in a competition binding assay. A. Cells were incubated with unconjugated CCL2 for 45 minutes at 37°C; then, conjugated anti-ACKR2 was added to determine levels of receptor binding inhibition by CCL2. ACKR2 bound to CCL2 with lower affinity, resulting in higher ACKR2 receptor binding with the anti-ACKR2 antibody compared to ACKR2-WT (ACKR2-V41A μ=88.5; ACKR2-WT μ=66.18; two-tailed T test; p-value 0.0153) (n=5; * = $p<0.05$). 100 minus the percentage of ACKR2+ cells were subtracted to determine the percentage of CCL2 blockage. ACKR2-V41A (μ=11.5) was blocked at a significantly lower percentage by CCL2 than ACKR2-WT (μ=33.82) (n=5; * = $p<0.05$).

Supplemental figure 3

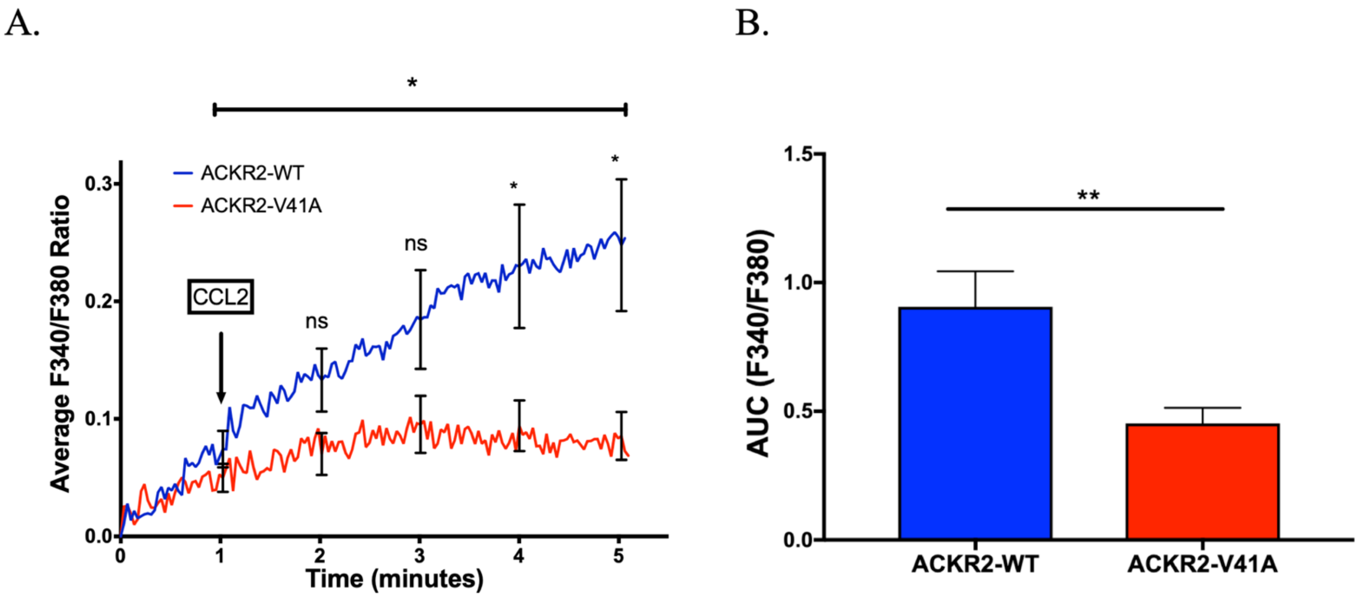

Supplemental figure 3. **ACKR2-V41A has lower levels of calcium signaling after activation with CCL2 ligand.** The calcium response of CHO-k1 cells with atypical chemokine ACKR2-WT and ACKR2-V41A receptors were measured. A. Calcium levels were measured after activation with CCL2 at the 1-minute time point. Cells expressing ACKR2-V41A had significantly lower Ca^{2+} influx than cells expressing ACKR2-WT starting at the 4 minutes time point (two tail T test, p-value 0.0052) (n=35; * = $p<0.05$). B. Area under the curved (AUC) analysis from minute 1 (CCL2 activation) to minute 5 (assay end point) confirmed that ACKR2-V41A had significantly lower levels of Ca^{2+} influx than ACKR2-WT (two tail T test, p-value 0.008) (n=35; ** = $p<0.01$).

Supplemental figure 4

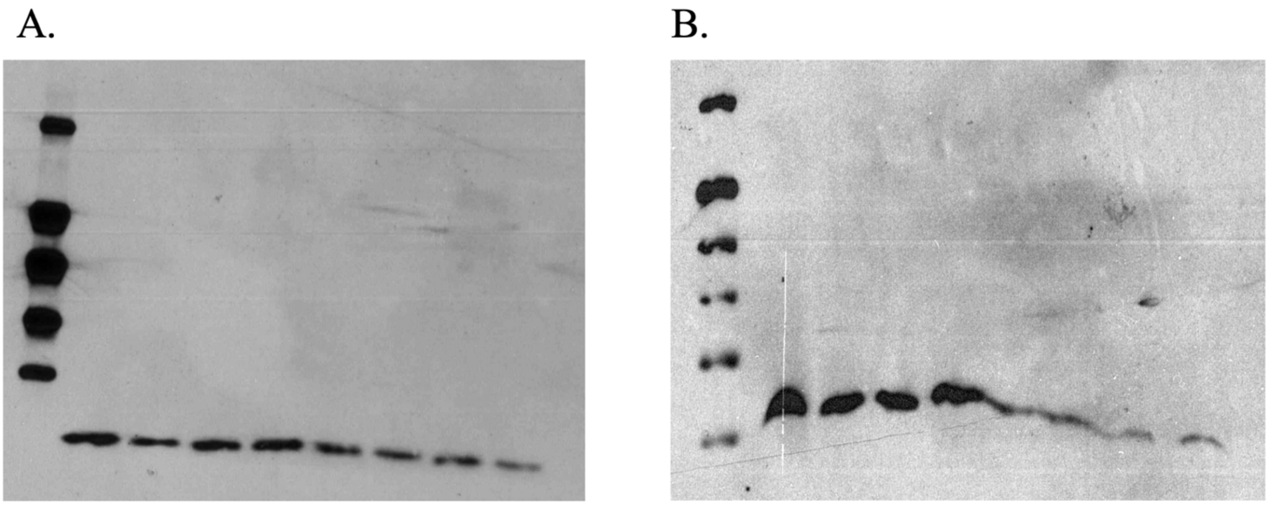

Supplemental figure 4. **Western blot cofilin and phosphorylated cofilin.** 300,000 CHO-k1 cells expressing either ACKR2-WT or ACKR2-V41A were seeded in a 6-well plates and serum-starved for 18 hours. Then, cells were stimulated with 100 nM of CCL2 and incubated at 37 °C in 5% CO_2 for 30 minutes. Cells were harvest, lysed using RIPA lysing buffer following manufacture recommendations. Lysed product was prepared for western blot assay. Two gels were run in parallel using the same lysed product samples. A. cofilin blot. B. phosphorylated cofilin blot.
